# Supplementary material for: The role of urban trees in reducing land surface temperatures in European cities
Source: Nat Commun. 2021 Nov 23;12:6763. doi: 10.1038/s41467-021-26768-w (PMC8611034; doi:10.1038/s41467-021-26768-w)
Supplement: Supplementary file 2 — Description of Additional Supplementary Files [file 41467_2021_26768_MOESM2_ESM.pdf]

Description of Additional Supplementary Files

File name: Supplementary Data 1
Description: All cities used for the analysis. Results from the city Prato have been excluded since the street tree information for this city was incomplete. In total 120.285 LANDSAT observations and 57.212 ASTER observations have been used.
