# Supplementary material for: The role of urban trees in reducing land surface temperatures in European cities
Source: Nat Commun. 2021 Nov 23;12:6763. doi: 10.1038/s41467-021-26768-w (PMC8611034; doi:10.1038/s41467-021-26768-w)
Supplement: Supplementary file 4 — Supplementary material [file 41467_2021_26768_MOESM4_ESM.pdf]

Supplementary Material

Supplementary Note 1: Data on urban trees (Copernicus Street Tree Layer) and high-resolution land-use of urban areas (Copernicus Urban Atlas) are available within the administrative boundaries of the following countries: *Albania, Austria, Bosnia and Herzegovina, Belgium, Bulgaria, Switzerland, Cyprus, Czech Republic, Germany, Denmark, Estonia, Spain (including Andorra), Finland, France, United Kingdom, Greece, Croatia, Hungary, Ireland, Isle of Man, Iceland, Italy, Liechtenstein, Lithuania, Luxembourg, Latvia, Montenegro, Republic of North Macedonia, Martinique, Malta, Northern Ireland, Netherlands, Norway, Poland, Portugal, Romania, Serbia, Sweden, Slovenia, Slovakia, Turkey, Kosovo.*

Supplementary Table 1: Response and predictor variables included in the process of fitting Generalized Additive Models.

Variables	Temporal coverage and resolution	Spatial resolution (unmodified)	Source
**Landsat-LST**	**01/01/2006 - 31/12/2018 (16-day)**	**30m**	**NASA/USGS**
**Aster-LST**	**01/01/2006 - 31/12/2018 (16 day)**	**90m**	**NASA/METI**
Y-coordinates (latitude)	-	Inherent to Landsat-LST & Aster-LST	Inherent to Landsat-LST/Aster-LST
X-coordinates (longitude)	-	Inherent to Landsat-LST & Aster-LST	Inherent to Landsat-LST/Aster-LST
Street tree layer	2012	~20m	Copernicus Land
Elevation (DEM)	-	100m	Copernicus
Aspect (ASP) – fraction of north facing slopes	-	100m	(based on DEM)
Urban atlas	2012	~20m	Copernicus land

Supplementary Table 2: Urban atlas LULC nomenclature describing the LULC in and around cities. Street tree data is only mapped in areas having an ID starting with 1 and being characterized as artificial surfaces.

<table>
<tr><td>ID</td><td>Description</td><td></td><td></td></tr>
<tr><td>11100</td><td>Continuous Urban fabric (S.L. > 80%)</td><td rowspan="6">Urban fabric</td><td rowspan="7">Artificial surfaces</td></tr>
<tr><td>11210</td><td>Discontinuous Dense Urban Fabric (S.L.: 50% - 80%)</td></tr>
<tr><td>11220</td><td>Discontinuous Medium Density Urban Fabric (S.L.: 30% - 50%)</td></tr>
<tr><td>11230</td><td>Discontinuous Low Density Urban Fabric (S.L.: 10% - 30%)</td></tr>
<tr><td>11240</td><td>Discontinuous very low density urban fabric (S.L. < 10%)</td></tr>
<tr><td>11300</td><td>Isolated Structures</td></tr>
<tr><td>12100</td><td>Industrial, commercial, public, military and private units</td><td>Industrial, commercial,</td></tr>
</table>

12210	Fast transit roads and associated land	public, military, private and transport units	
12220	Other roads and associated land		
12230	Railways and associated land		
12300	Port areas		
12400	Airports		
13100	Mineral extraction and dump sites	Mine, dump and construction sites	
13300	Construction sites		
13400	Land without current use		
14100	Green urban areas	Artificial non-agricultural vegetated areas	
14200	Sports and leisure facilities		
21000	Arable land (annual crops)	Agricultural areas, semi-natural areas and wetlands	Agricultural areas, semi-natural areas and wetlands
22000	Permanent crops		
23000	Pastures		
24000	Complex and mixed cultivation patterns		
25000	Orchards		
31000	Forests	Forests	Forests
32000	Herbaceous vegetation associations		
33000	Open spaces with little or no vegetation		
40000	Wetlands	Water	Water
50000	Water		

Supplementary Table 3: Different model set-ups. The column with grey background denotes the model selected to show the results in the main part of the manuscript (see Supplementary Note 2). The basis dimension for each thin plate regression spline was set to 5 (i.e. k=5).

Variable/Term	Model 1 (Landsat)	Model 2 (Aster)	Model 3 (Landsat)	Model 4 (Landsat)	Model 5 (Landsat)	Model 6 (Landsat)
x,y (lat/lon)	2d-tensor (k=5)	2d-tensor (k=5)	2d-tensor (k=10)	-	2d-tensor (k=5)	2d-tensor (k=10)
Urban trees	Thin plate	Thin plate	Thin plate	linear	Thin plate	Thin plate
Elevation	Thin plate	Thin plate	Thin plate	linear	Thin plate	Thin plate
Aspect	Thin plate	Thin plate	Thin plate	linear	Thin plate	Thin plate
LULC, green spaces	Thin plate	Thin plate	Thin plate	linear	Thin plate	Thin plate
LULC, pasture	Thin plate	Thin plate	Thin plate	linear	-	-
LULC, forest	Thin plate	Thin plate	Thin plate	linear	-	-
LULC, others (artificial)	Thin plate	Thin plate	Thin plate	linear	Thin plate	Thin plate
LULC others, (water etc.)	Thin plate	Thin plate	Thin plate	linear	-	-
Extent	All	All	All	Only Artificial urban surfaces	Only Artificial urban surfaces	Only Artificial urban surfaces

Supplementary Table 4: Definition of the LULC types that were used to calculate LST differences (adapted from Copernicus [1]).

LULC type	Description
Continuous urban fabric	Land cover:<br>Degree of soil sealing > 80%<br>Built-up areas and their associated land. Buildings, roads and sealed areas cover most of the area; non-linear areas of vegetation and bare soil are exceptional<br><br>Land use:<br>Predominant residential use: areas with a high degree of soil sealing, independent of their housing scheme (single family houses or high rise dwellings, city centers or suburb).
Urban trees/Street tree layer	The Street Tree Layer (STL) includes contiguous rows or patches of trees covering 500m² or more and with a minimum width (MinMW) of 10 m over Artificial surfaces (nomenclature class 1 of the urban atlas) inside urban areas (i.e. rows of trees along the road network outside urban areas or forest adjacent to urban areas should not be included).
Urban green spaces/Green urban areas	Public green areas for predominantly recreational use such as gardens, zoos, parks, castle parks and cemeteries. Suburban natural areas that have become and are managed as urban parks. Forests or green areas extending from the surroundings into urban areas are mapped as green urban areas when at least two sides are bordered by urban areas and structures, and traces of recreational use are visible.
Pastures	Pasture and meadow under agricultural use, grazed or mechanically harvested. Wooded meadows. (Not included are fields under crop rotation systems).

Forests	Broad leaved forest, coniferous forest and mixed forest; Transitional woodland and shrub (clear cut, new plantations and regeneration, or damage forest); With ground coverage of tree canopy > 30%, tree height > 5 m, including bushes and shrubs at the fringe of the forest; Included are plantations such as Populus plantations, Christmas tree plantations; Forest regeneration / re-colonization: clear cuts, new forest plantations. Not included are: Forests within urban areas and/or subject to high human pressure.

Supplementary Fig. 1: a) Boxplot showing the difference in the cooling provided by urban trees during hot extremes ($\Delta T[K]_{HE}$) and the cooling provided during average summertime conditions ($\Delta T[K]_{JJA}$). Significance levels (0.1-0.05 (.), 0.05-0.01 (*), 0.01-0.001 (**) and 0.001-0 (***)) indicate whether the median is significantly different from zero (based on a Wilcoxon signed-rank test). b) The map shows the difference in the cooling provided by urban trees during hot extremes and the cooling provided during average summertime conditions for each city. In addition, a spatially smoothed trend of these differences is added as a background to the map. The size of the dots indicates the uncertainties in the form of standard errors (SE).

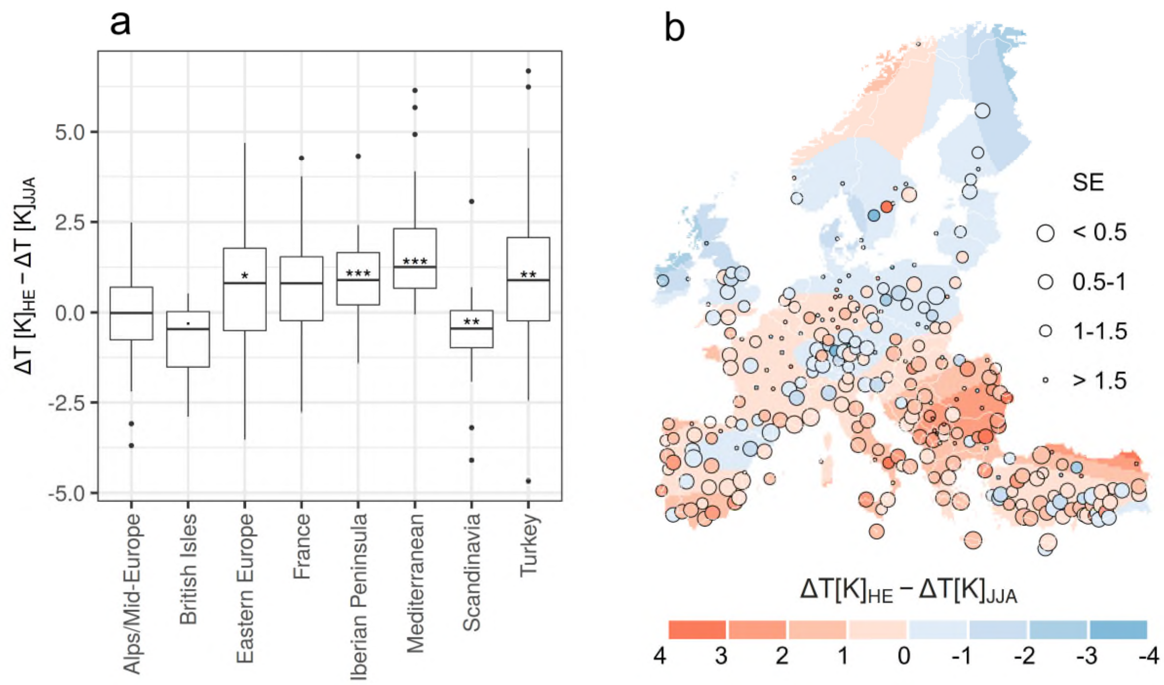

Supplementary Fig. 2: a) Correlation of temperature difference Urban Trees (UT) – continuous Urban Fabric (UF) with temperature difference Forest (F) – continuous Urban Fabric (UF) for average summer conditions b) Correlation of temperature difference Urban Trees – continuous Urban Fabric with temperature difference Forest (F) – continuous Urban Fabric (UF) for the hottest observation. c) Correlation of temperature difference urban Green Spaces (GS) – continuous Urban Fabric (UF) with temperature difference “Pastures (P) – continuous Urban Fabric (UF) for average summer conditions d) Correlation of temperature difference urban Green Spaces (GS) – continuous Urban Fabric (UF) with temperature difference Pastures (P) – continuous Urban Fabric (UF) for the hottest observation.

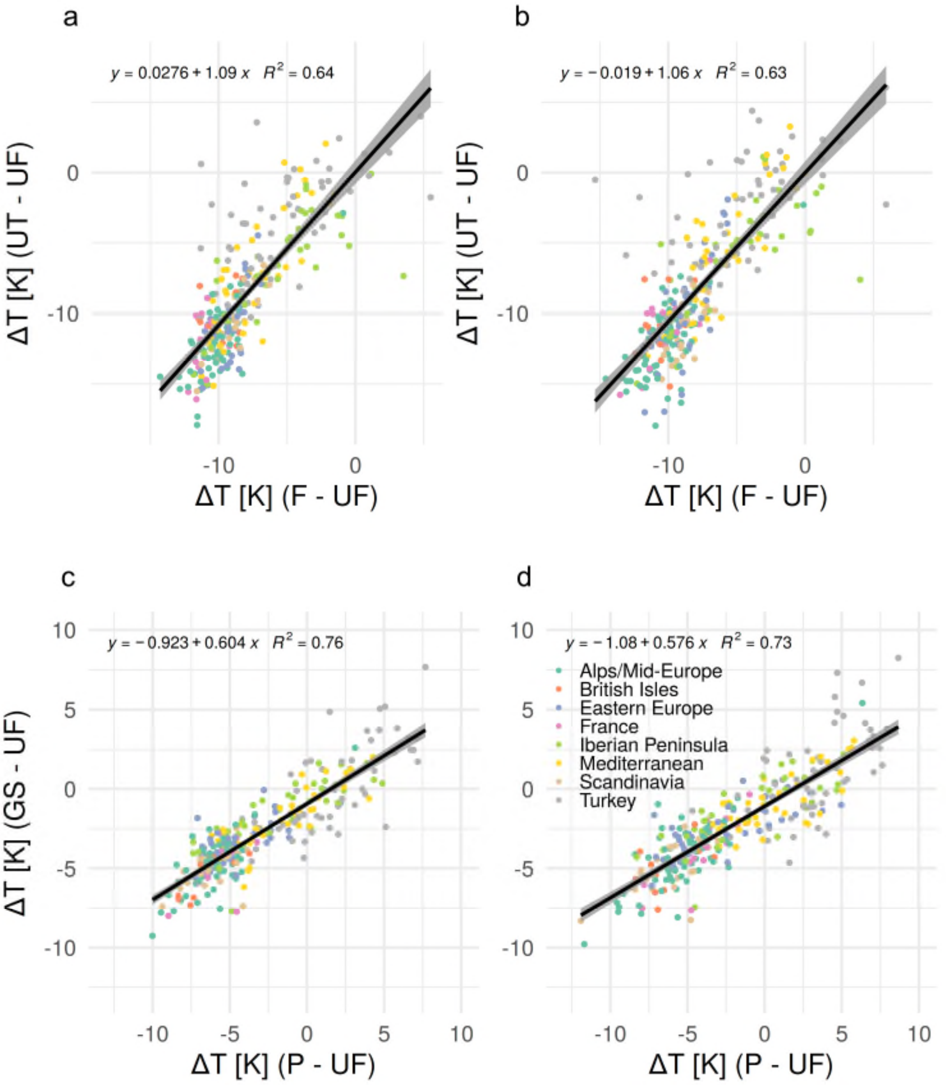

Supplementary Fig. 3: Smoothed temperature differences between urban trees and urban fabric (UT-UF), forests and urban fabric (F-UF), green spaces and urban fabric (GS-UF) and pastures minus urban fabric (P-UF).

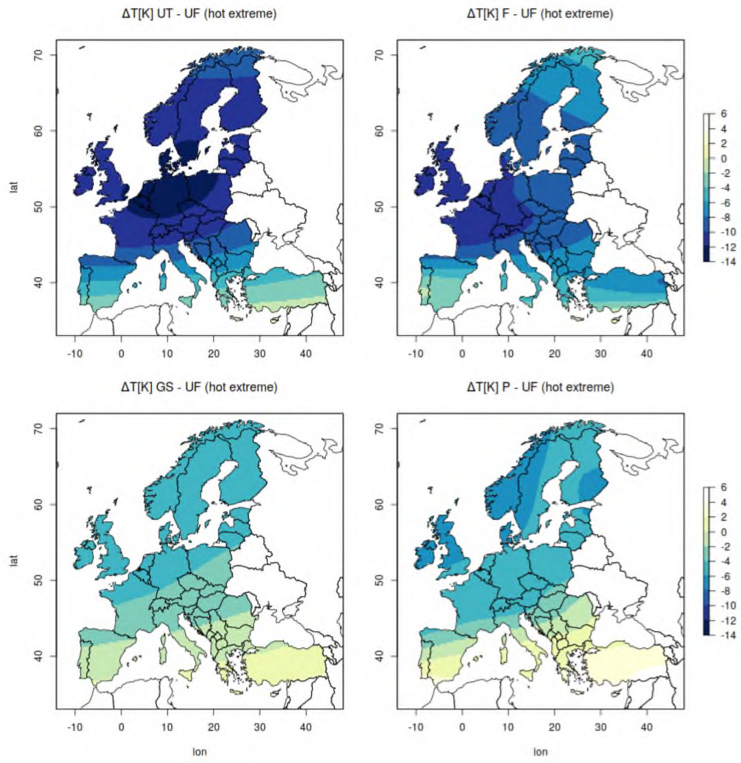

Supplementary Fig. 4: a) Albedo differences between urban and forested areas. b) Albedo of forested areas. c) Albedo of continuous urban fabric. Outliers of albedo values have been removed in all three figures (i.e. in figure a values below -0.25 and above 0.25, in figure b/c values below 0.0 and above 0.3). Black dots indicate the mean albedos (and mean albedo differences) in each region.

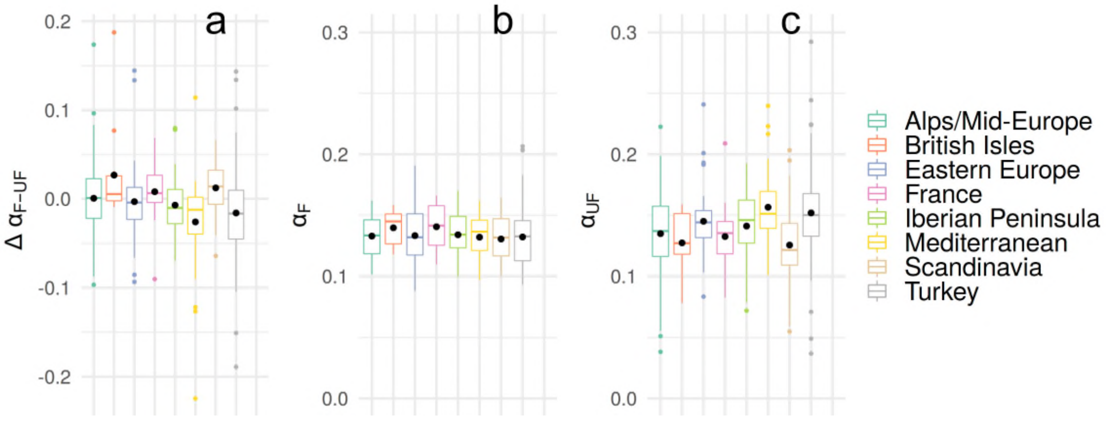

Supplementary Fig. 5: a) Evapotranspiration (ET) estimates over forests for cities in different regions. b) Evapotranspiration (ET) estimates over pastures for cities in different regions. Black dots indicate the mean ET values in each region.

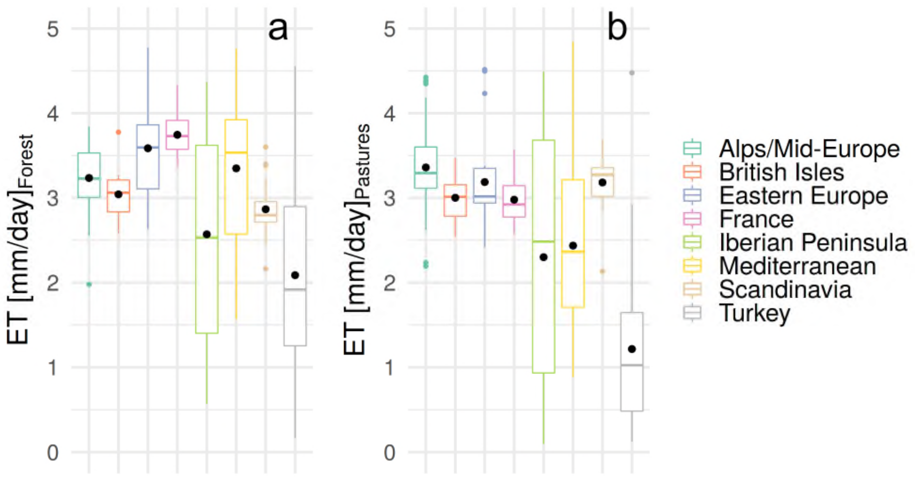

Supplementary Fig. 6: Comparison of Copernicus Street Tree Layer (STL) and tree height mapped for a part of the city of Basel (Switzerland) based on Lidar [2]. The Street Tree Layer (STL) indicates the location of contiguous urban patches. The Lidar data shows individual trees and their height. A lot of single trees and thin rows of trees are not included in the STL data. The STL indicates some larger forest patches (lower left in the map), where the Lidar data suggests that there is very little tree cover.

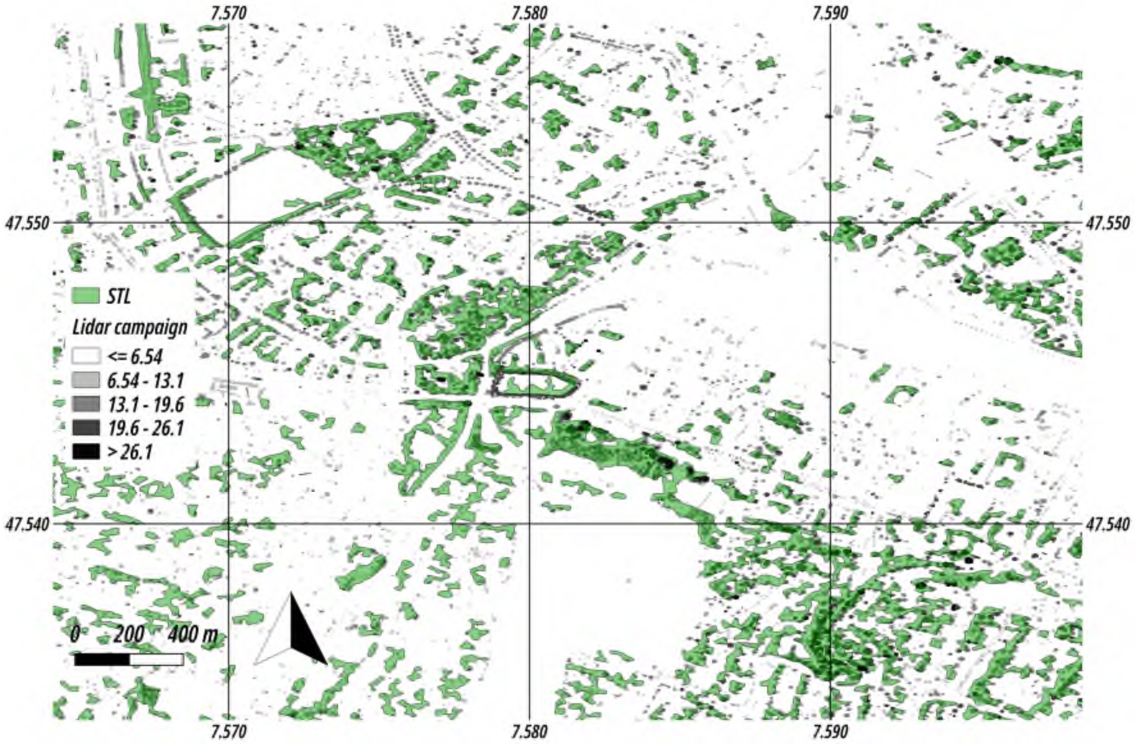

Supplementary Fig. 7: Comparison of Copernicus Street Tree Layer (STL) and google earth image of a part of Barcelona (Spain). The map shows again that single trees and thin rows of trees are not included in the STL data. It also shows that some larger forest patches may be missing in the STL data.

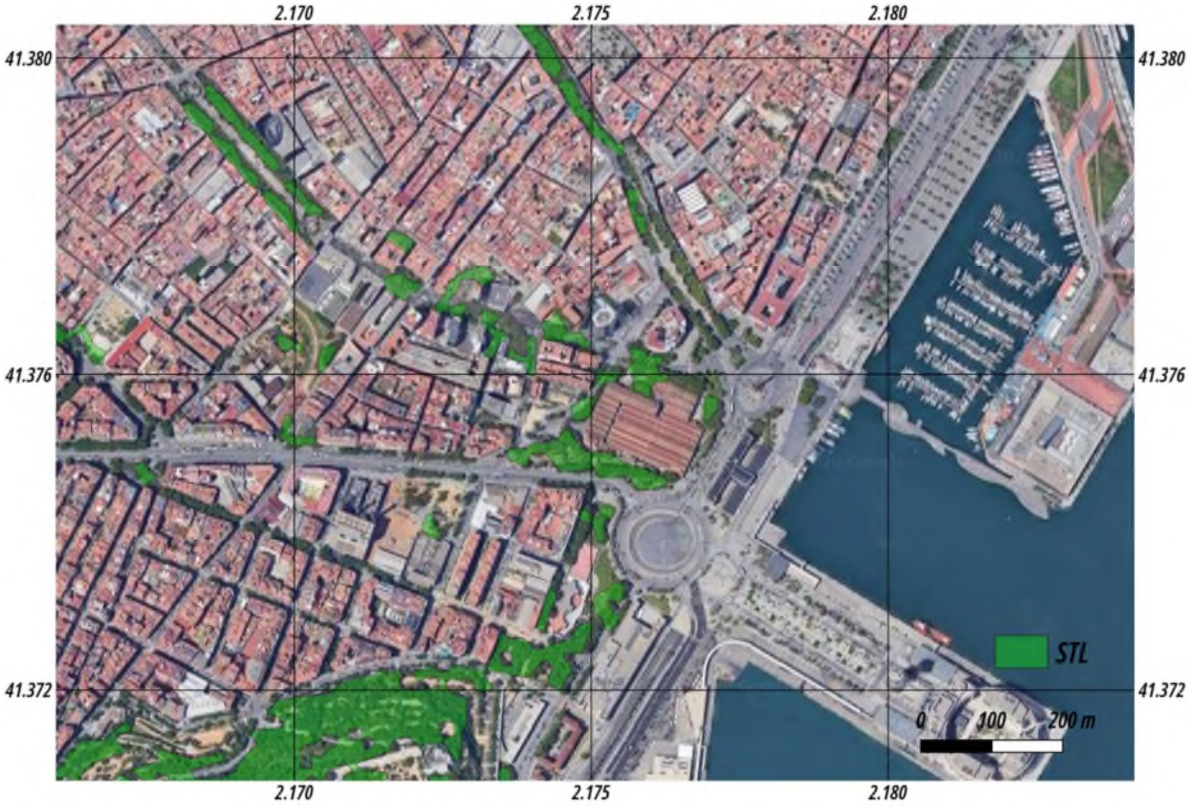

Supplementary Fig. 8: Influence of different emissivities chosen for the Landsat LST product. The temperature differences between urban trees and continuous urban fabric are very similar for all emissivities. The NDVI-based emissivity seems to lead to a slightly higher temperature difference than the other emissivities.

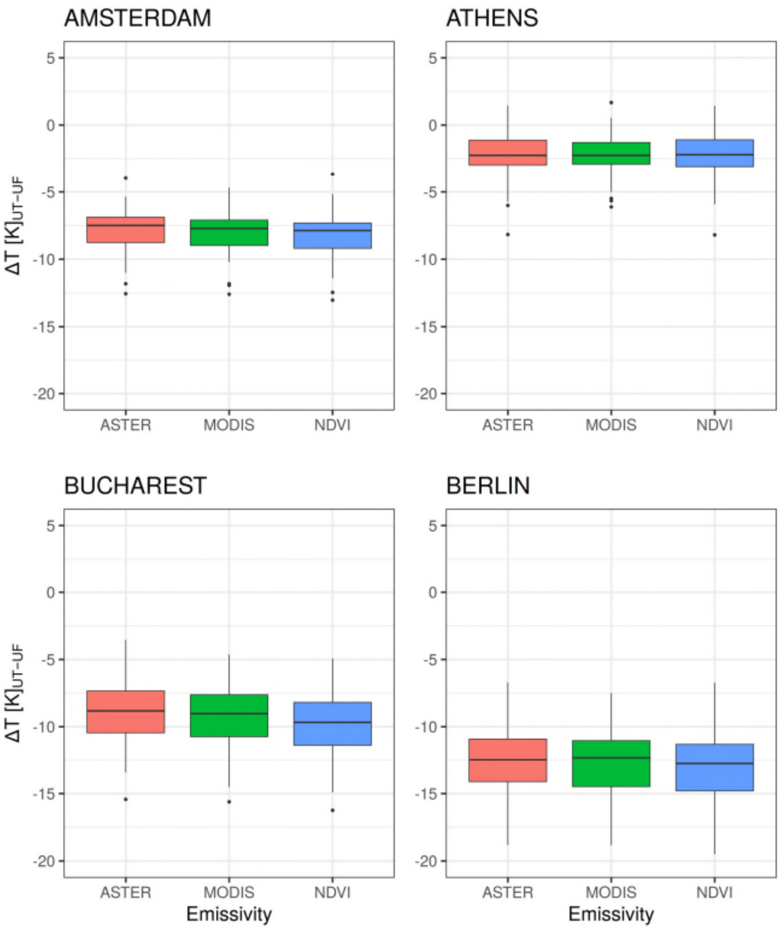

Supplementary Fig. 9: Correlation between average summer temperature differences (between urban trees and continuous urban fabric) estimated based on LANDSAT data and temperature differences based on ASTER data.

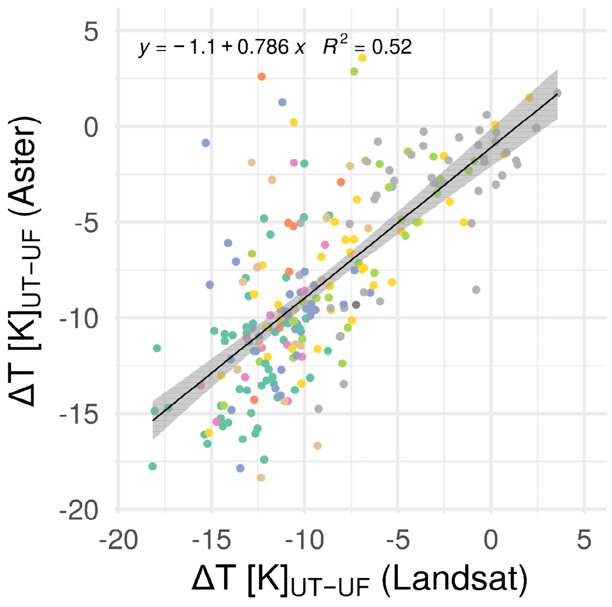

Supplementary Fig. 10: The coefficient of determination R^2 of all calibrated models in different regions

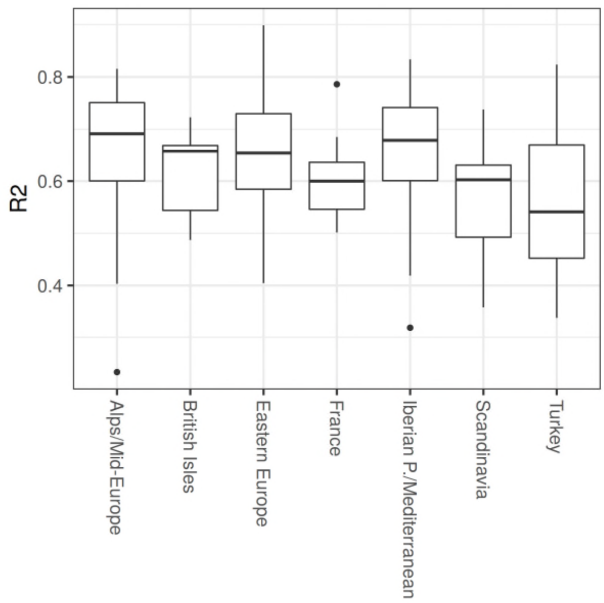

Supplementary Note 2: Additional validation and discussion of modelling approach

Results obtained with different modelling set-ups showed in general good agreement (Supplementary Fig. 11 and Supplementary Fig. 12). We tested the sensitivity of (1) changing the study perimeter of each city, of (2) using different model parameters and of (3) using the Aster data instead of the Landsat data. First, we focused on testing the sensitivity of changing the perimeter of each city. In model 1-3 we included all data available (including the surrounding of cities), whereas in model 4-6 we only included areas that are defined as artificial surfaces (Supplementary Table 2). The agreement between model 1 that includes all data available for each city and model 5-6 (including smooth functions) with a restricted amount of data used, are closely correlated (Supplementary Fig. 11). Second, we focused on choosing different modelling parameters. The basis dimension for the 2d-tensor product smooth of the interaction of x and y coordinates was changed from 25 (k=5) to 100 (k=10). This hardly had an effect on the models that were fitted for only artificial urban areas, but it had an effect on models fitted for all data available, which can be seen by comparing the results of model 1 and 3. The change in the basis dimension of the 2d- tensor smooth seemed especially relevant in Scandinavia, where the predicted temperature difference between urban trees and continuous urban fabric decreased when increasing the basis dimension of the 2d-smooth. This indicates that in some cities the basis dimension of the 2d-smooth was restrictively low. On the other hand, we observed that the LULC effect in some cities decreased towards zero if the basis dimension was not restricted. We show the results of using different basis dimensions for the 2d-smooths, but are aware that selecting a basis dimension individually for each city could be preferable to choosing a constant basis dimension for each city. An additional change in the model parameters was to model the contribution of each predictor in a linear way and to not include smooth functions. The main difference between model 1 (including smooth function) and model 4 (only linear) can be seen in the regions Iberian Peninsula/Mediterranean and Turkey. The linear model (4) predicts larger temperature differences between urban trees and urban fabric than model 1. The correlation between the two models is still quite high (Pearson correlation coefficient: 0.62). However, the linear model shows a lower R2, which may indicate that there are significant non-linearities that would not be captured in such a model. A comparison between the Aster- and Landsat-based analysis shows that the analysis based on Aster predicts lower temperature differences between urban trees and continuous urban fabric in Scandinavia (model 2). Since there are a lot less Aster-observations available, particularly during hot extremes, the uncertainties of the observations strongly increase (indicated by a very large interquartile range of the boxplot in Scandinavia). Thus, the median temperature difference between Aster- and Landsat-based results in Scandinavia may not be systematic, but rather a product of noisy observations.

Supplementary Fig. 11: Boxplots of temperature differences between urban trees and urban fabric for different model set-ups and different regions (more details on model set-ups Supplementary Table 3). Boxes show the first and third quartile; whiskers show the largest/smallest values, but do not extend beyond 1.5 times of the interquartile range; outliers are shown as separate points.

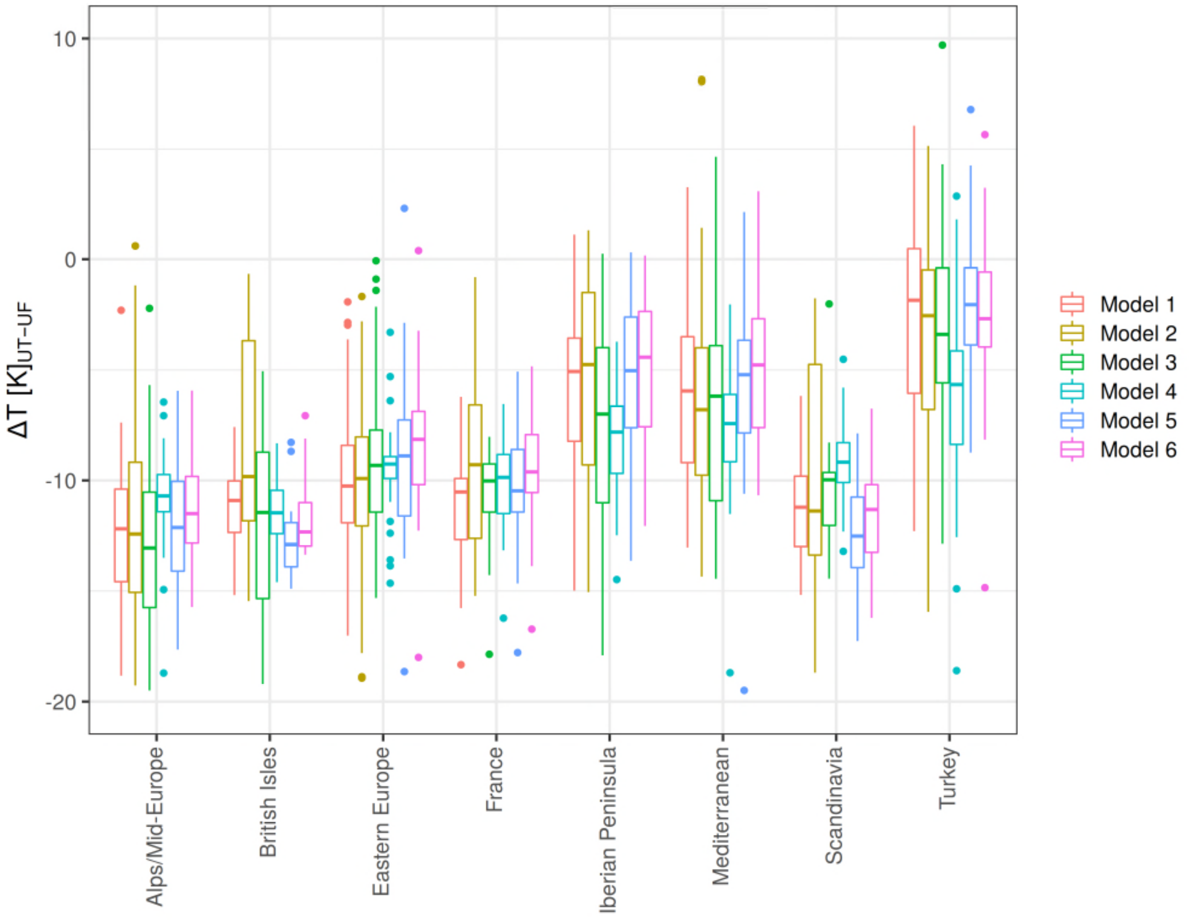

Supplementary Fig. 12: Matrix of scatterplots to visualize the agreement of temperature differences between urban trees and urban fabric predicted with different models (Supplementary Table 3). The panels above the diagonal show the Pearson correlation coefficient. The panels below the diagonal show the scatterplots with a smooth based on locally weighted polynomial regression (LOWESS). Model 1 is the model that has been selected to display results in the main part of the manuscript. It shows on average the highest correlation with all other models.

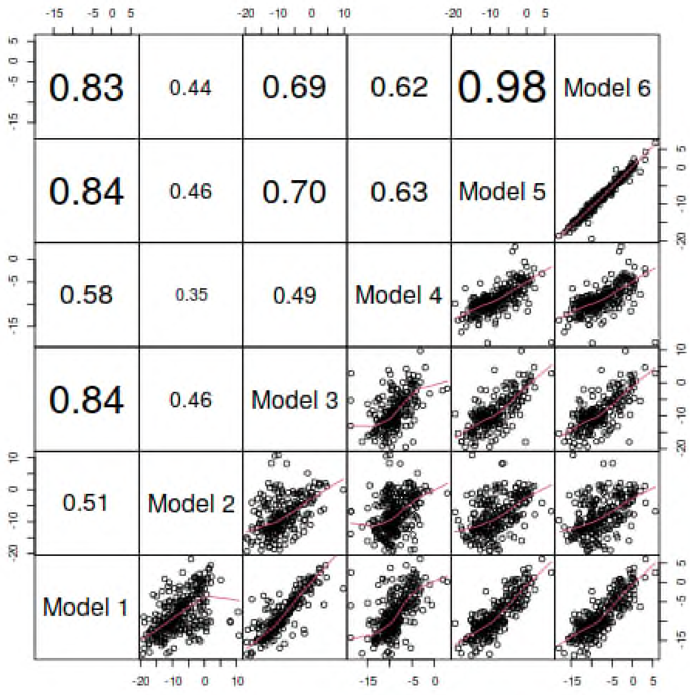

Supplementary Note 3: Data gaps due to cloud cover and low sampling frequency.

Due to seasonal variations in cloud-cover, there are more Landsat observations available in summer than in winter (Supplementary Fig. 12 a, b). The number of observations in winter is particularly low for cities in Scandinavia and comparably high for cities of Turkey, the Iberian Peninsula and the Mediterranean. This increases the uncertainty in winter, but is of minor importance since we are focusing on summertime temperatures. To better understand potential sampling biases in summer, we used E-OBS (v22.0e) temperature data [3], which is a gridded dataset of air temperature based on station data and available for all conditions. By separating all E-OBS observations for each city into quantiles, we separated dates when low temperatures were observed from days when hot temperatures were observed. These dates were matched with the dates, when Landsat observations were available, to find out whether Landsat data were missing for certain quantiles. The regionally summarized results show that for cities in all regions observations are available for low and high quantiles in summer (Figure 12 c). However, in some regions (e.g. France and British Isles) there are much less observations available for low quantiles than in other regions (e.g. Turkey). We also tested whether LST data was more often available for certain LULC types than for others, but did not find substantial differences (Figure 12 d).

Cloudiness clearly leads to data gaps and highlights that our results should be strictly interpreted as being only valid for cloud-free conditions. Since observations for high temperatures are consistently and frequently available for all regions, the smooth functions that are used to estimate temperature differences between different LULC types, are relatively robust and it is unlikely that we strongly confound temporal and spatial effects. Missing data for colder and

presumably cloudy days in summer will, however, have an impact on average summertime temperature differences between different land-covers. Since the differences in the amount of observations during hotter and colder days in summer are relatively small in Scandinavia, Turkey, Iberian P./Mediterranean, it can be assumed that our observations would allow relatively robust conclusions about the full/true climatology in these regions. In regions such as the British Isles, France, Eastern Europe and Mid-Europe, including temperature differences during cloudy days (which are presumably small) will have an important impact on summertime mean temperature differences between different LULC types and hence may cause some bias.

Supplementary Fig. 13: Analysis of temporal and spatial sampling biases. a) Number of observations summarized for different European regions and four seasons (DJF, MAM, JJA and SON). b) Number of observations available in each month. c) Average number of days with Landsat observations available for each E-OBS quantile in summer. The first quantile is abbreviated as Q_JJA_1, the second one as Q_JJA_2, …, and the last as Q_JJA_10. d) Fraction of each land-cover type that is observed in different regions.

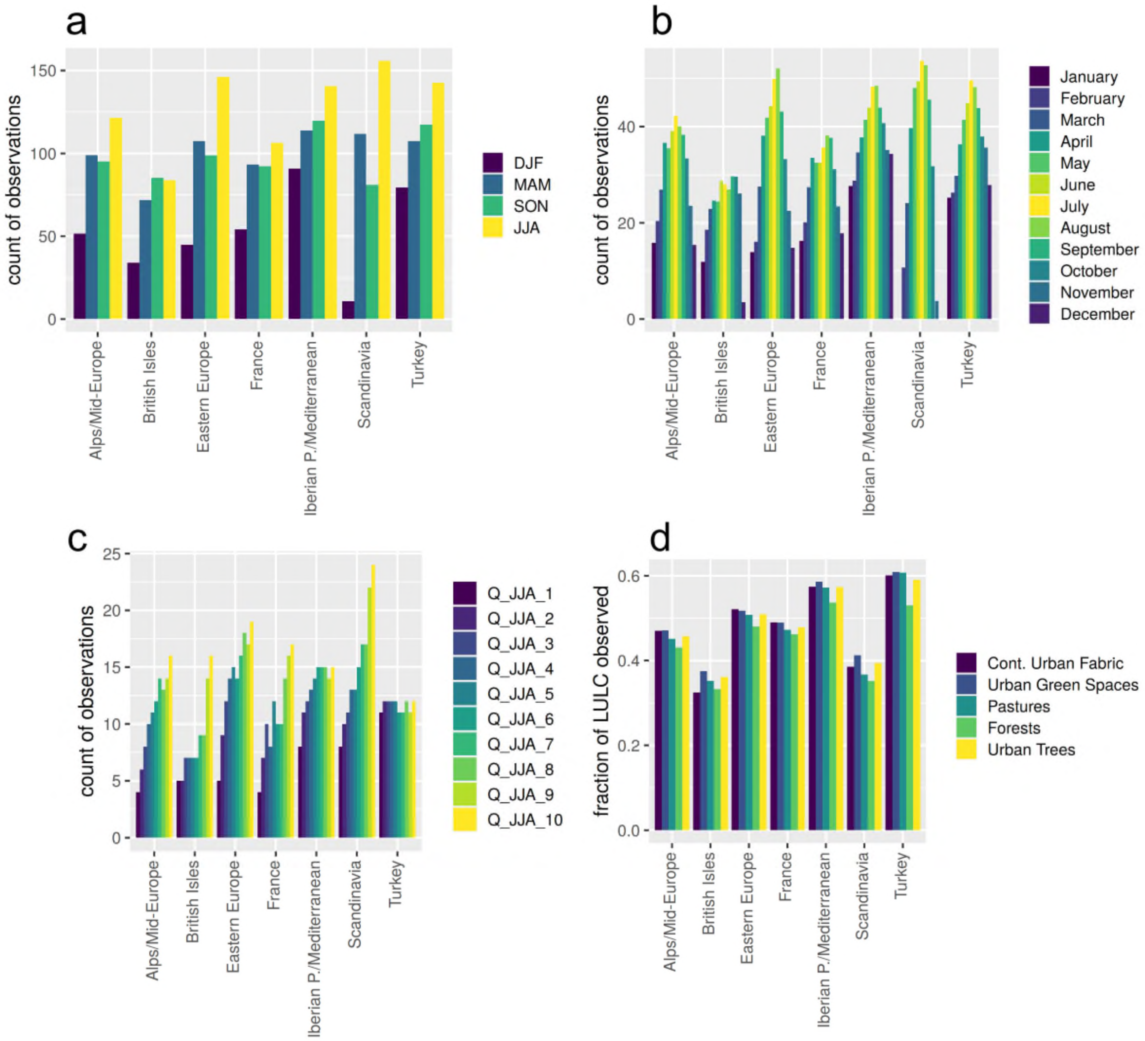

Supplementary Note 4: Albedo value uncertainties.

Albedos of continuous urban fabric in different regions were calculated separately for white- and black-sky albedo (WSA/BSA). WSA values are generally higher, however, we find that the regional patterns are the same for WSA and BSA (Supplementary Fig. 14). Since BSA and WSA show very similar regional trends, it seems rather unlikely that blue-sky albedo values will strongly deviate from these trends. However, if diffuse radiation over cities in certain European regions is much larger (e.g., due to air pollution) than in other regions, there may be slight changes in these trends, compared to the ones we see when weighing WSA and BSA equally.

Supplementary Fig. 14: Black Sky Albedo (BSA) and White Sky Albedo (WSA) of continuous urban fabric in different European regions.

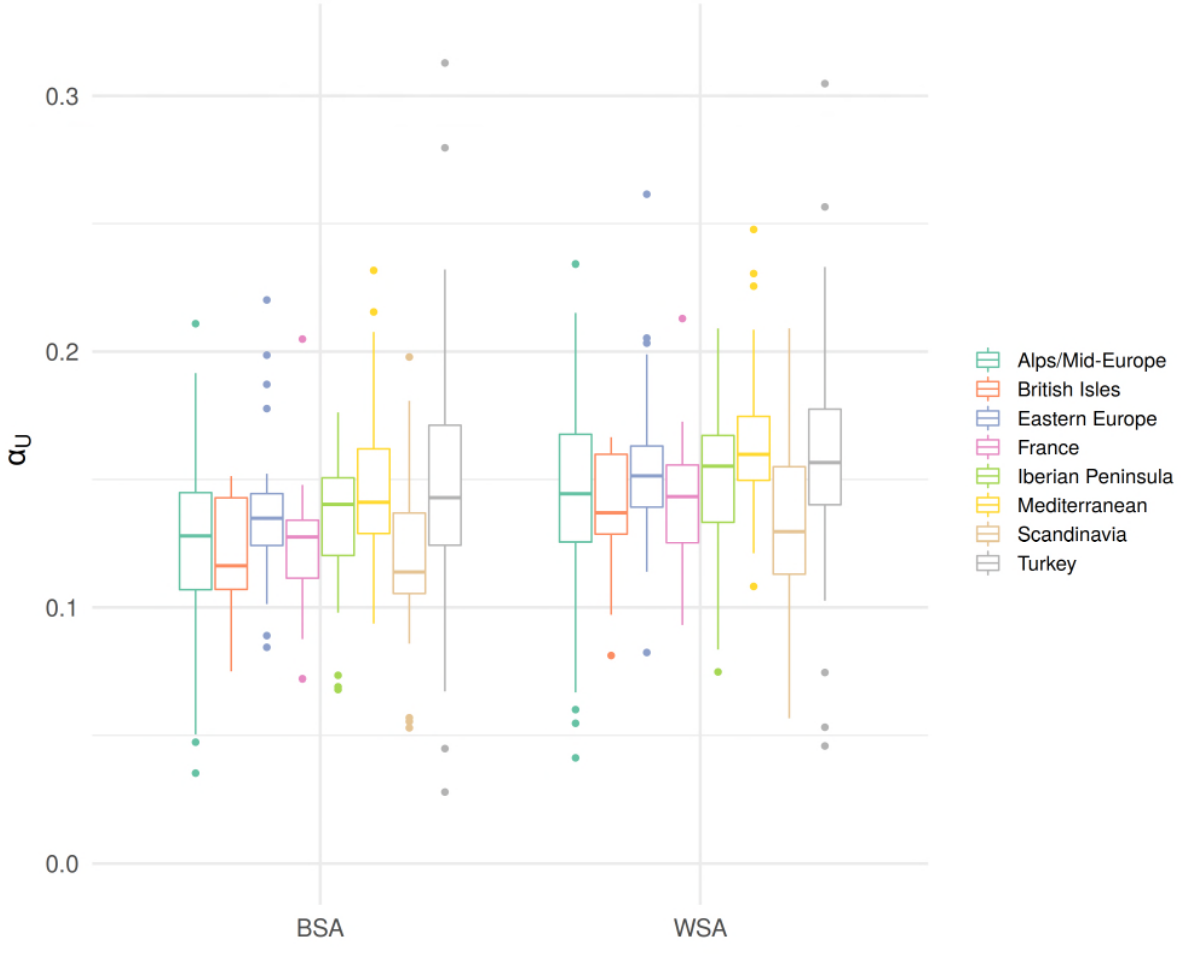

Supplementary Note 5: The potential of trees to reduce temperatures depends on morphological characteristics of the urban fabric in their surroundings. For example, it has been shown that the cooling effect of trees is masked by shading in deep street canyons [4]. To further test the influence of urban morphology on our results, we included data on building height provided by Copernicus (https://land.copernicus.eu/local/urban-atlas/building-height-2012) for the capitals of Europe. The results on the LST difference between urban trees and urban fabric in different European cities was not significantly influenced when including building height as a predictor variable (Supplementary Fig. 16). One reason for this could be that the different categories of urban fabric that we include in

the model are quite strongly related to the building height (Supplementary Fig. 16) and hence there is not that much new information added. This is in line with studies [5, 6, 7] showing that the influence of 2D variables (e.g., the amount of soil sealing) are often more relevant than 3D variables (e.g., building height). On the other hand, in particular during nighttime, the influence of 3D variables may be very important [8].

Supplementary Fig. 15: Boxplots of building heights for each category of urban fabric in the Copernicus urban atlas. Dense urban fabric categories are associated with higher buildings.

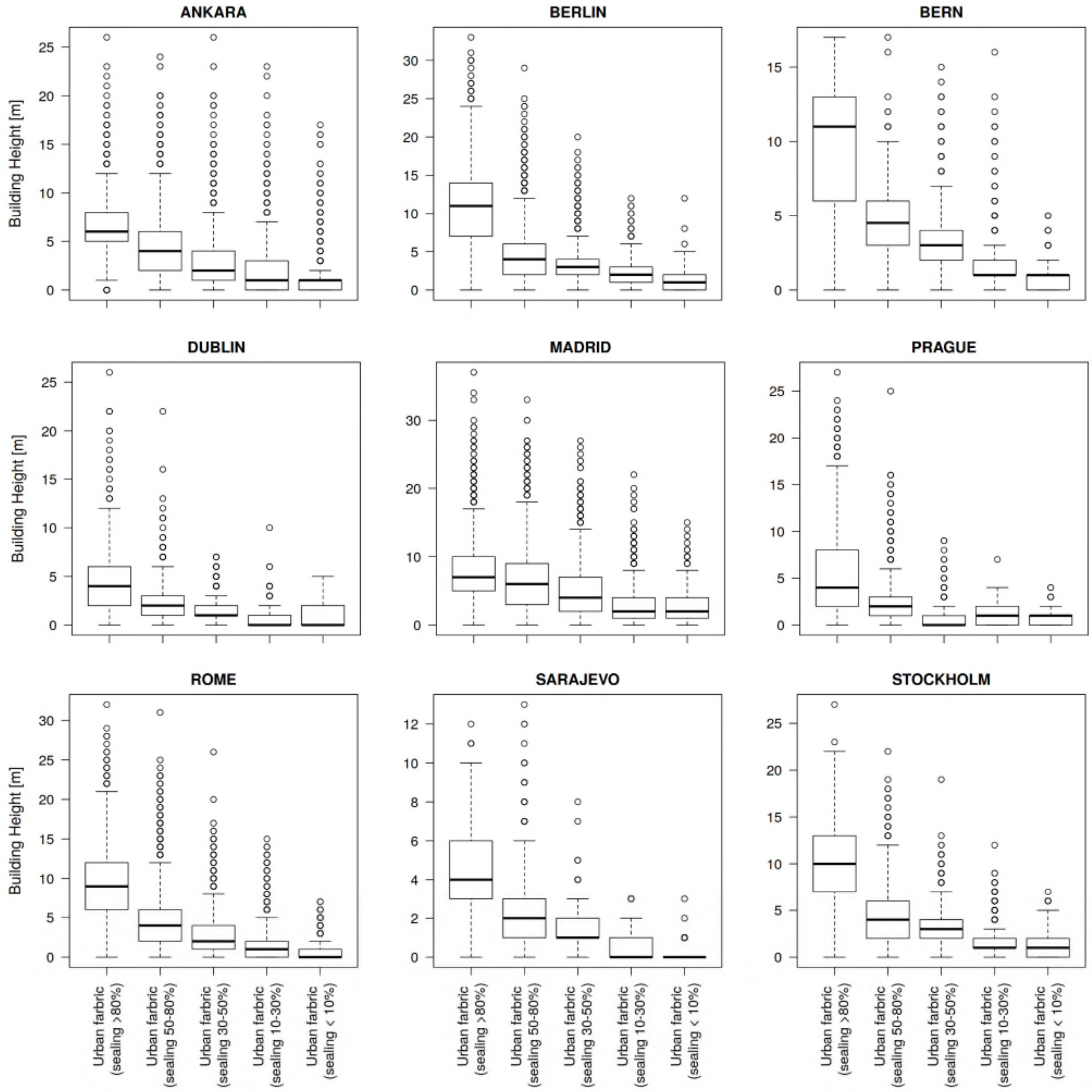

Supplementary Fig. 16: Correlation between temperature differences (urban trees – minus continuous urban fabric), when building height is included as an additional predictor variable. When building height is included in the model fit, the prediction of temperature differences between urban trees and continuous urban fabric includes average values of building height in areas where we find continuous urban fabric and a building height of zero for urban tree areas.

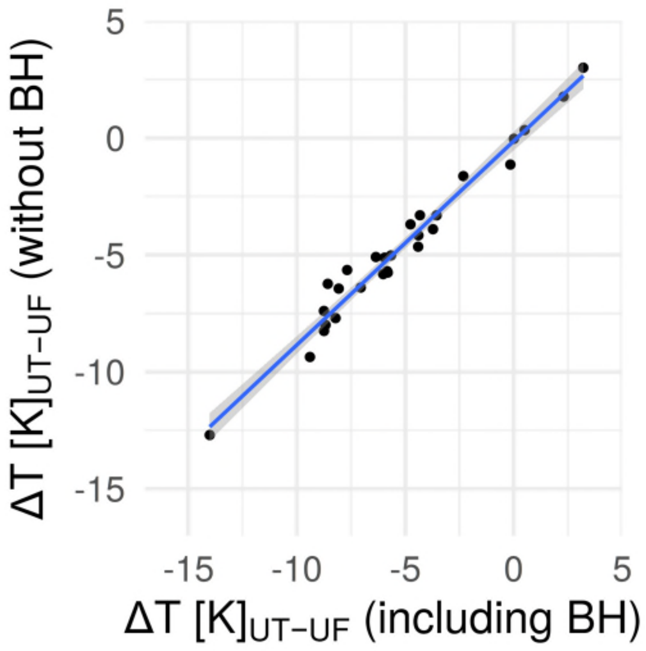

Supplementary Fig. 17: Comparison of temperature differences between urban vegetation (trees and treeless greenspaces) and continuous urban fabric during average summertime (JJA) conditions and during hot extreme conditions (UT = Urban Trees, UF = continuous Urban Fabric, GS = treeless urban Green Spaces).

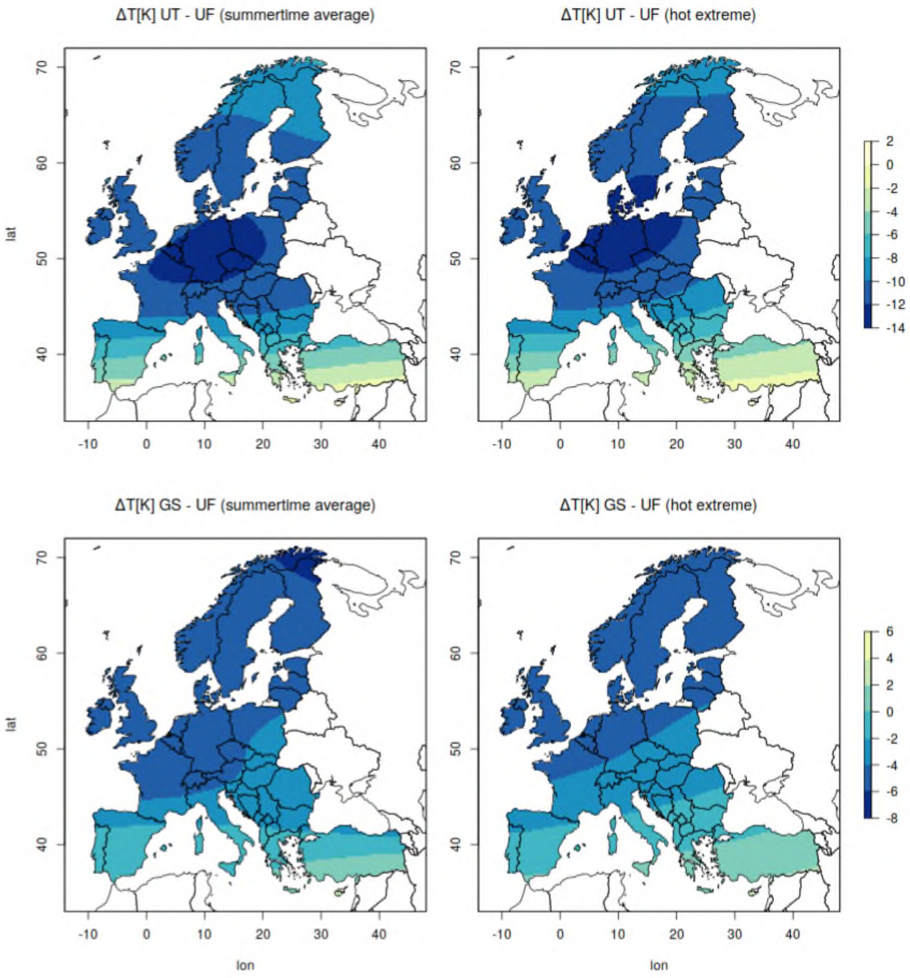

Supplementary Fig. 18: LST differences between urban trees and continuous urban fabric for different background temperatures shown exemplary for 20 randomly selected European capitals. The blue lines indicate a smooth function that is fitted to approximate these differences based on background temperature. Uncertainties are indicated in the form of a confidence interval.

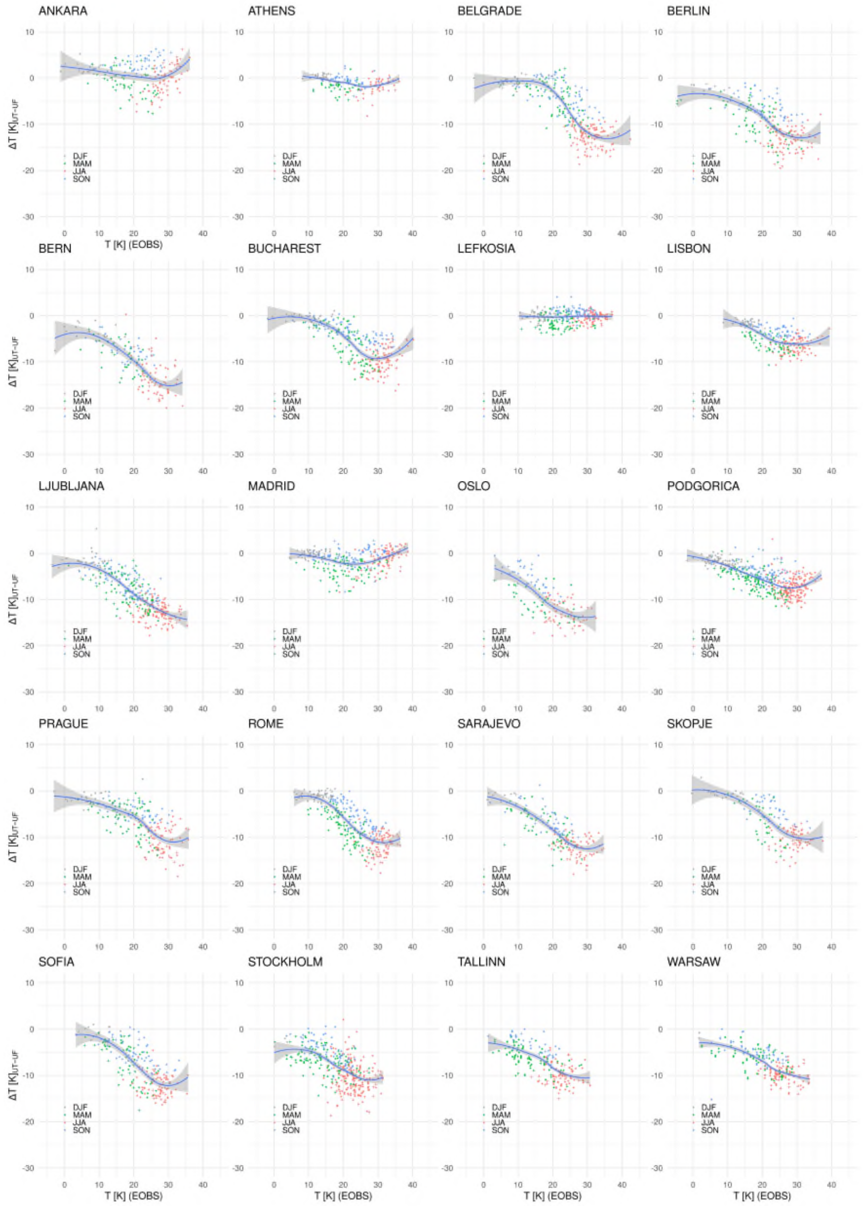

Supplementary Fig. 19: Scatterplots of background temperatures and albedo values of urban areas (left) and forested areas (right).

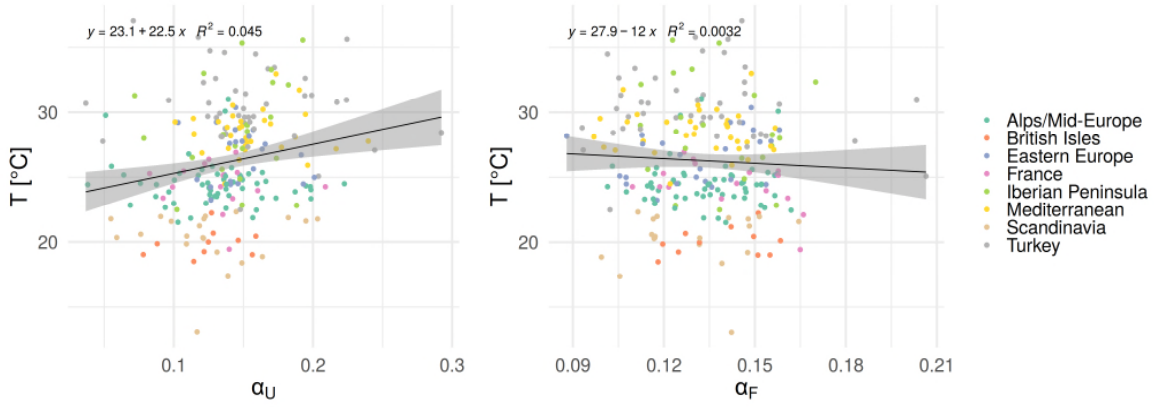

Supplementary Fig. 20: Fraction of artificial urban surfaces covered by trees and fraction of artificial urban surfaces covered by green spaces.

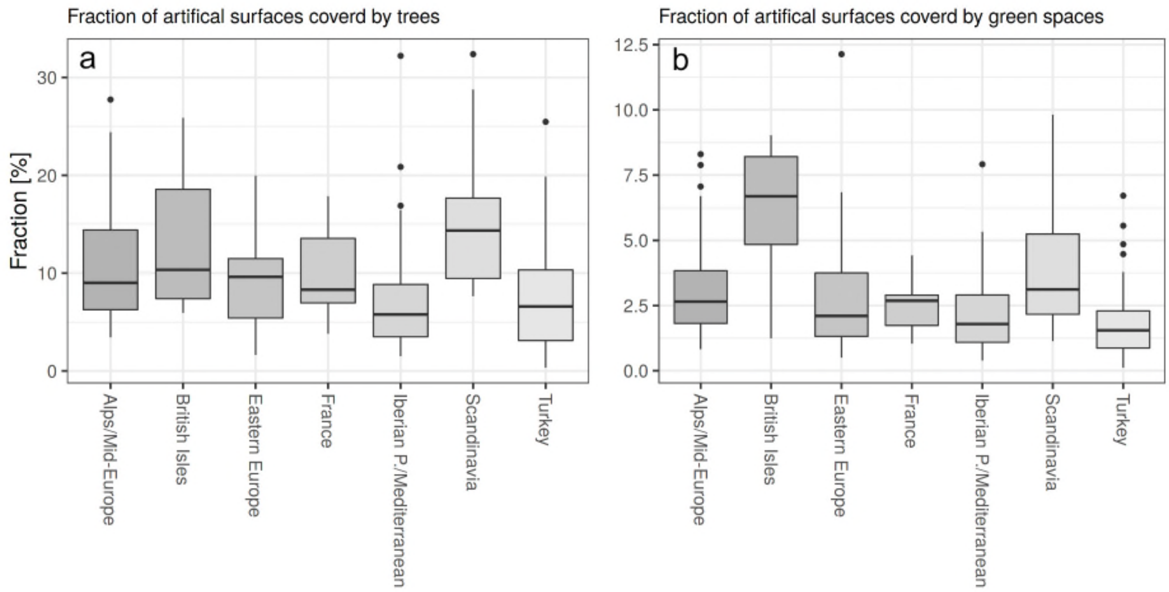

Supplementary Fig. 21: LST differences between urban trees and urban fabric based on NDVI emissivity plotted against LST differences between urban trees and urban fabric based on MODIS and ASTER emissivity.

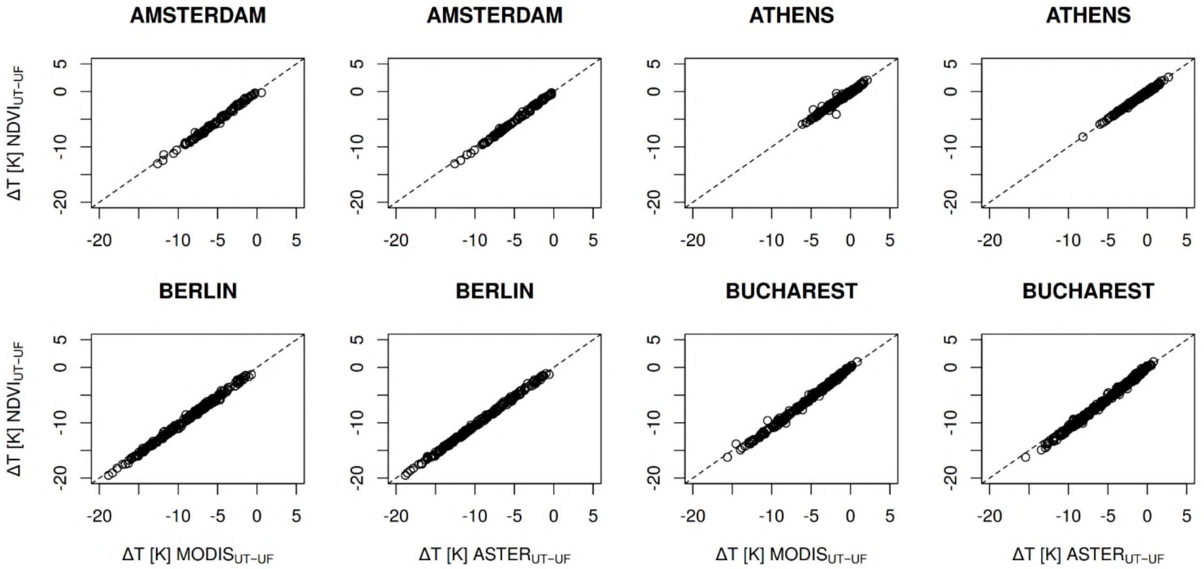

Supplementary Fig. 22: Correlation and differences between black- and white-sky albedo of European cities. a) Correlation between black- and white-sky albedo of urban fabric. b) Differences between black- and white-sky albedo of urban fabric. c) Correlation between black- and white-sky albedo of forests. d) Differences between black- and white-sky albedo of urban fabric.

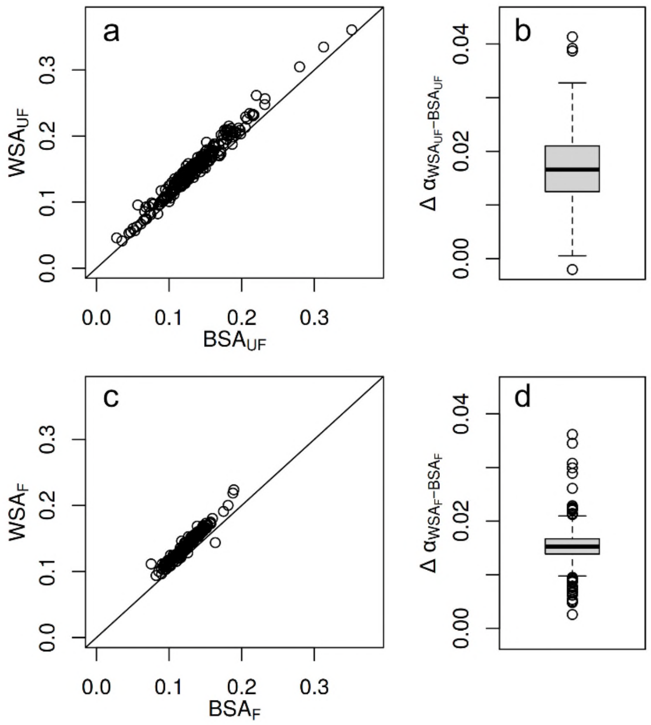

Supplementary Fig. 23: Correlations between LST differences (urban trees minus urban fabric) and albedo differences (forests minus urban fabric). The albedo differences are calculated as $\Delta\alpha_{F-UF} = \alpha_F - \alpha_{UF}$ with $\alpha_F = \omega_F\, \alpha_{F,WSA} + (1-\omega_F)\, \alpha_{UF,BSA}$ and $\alpha_{UF} = \omega_{UF}\, \alpha_{UF,WSA} + (1-\omega_{UF})\, \alpha_{UF,BSA}$, where ω can be understood as the weight given to white- and black-sky albedo or as the ratio of the surface downward diffuse shortwave radiation to the surface downward total shortwave radiation [9]. The weights (i.e. ratios) ω_F and ω_{UF} were varied in several ways between 0 and 1 to test how these choices influence the correlation between LST and albedo. Regions are numbered in the following way: Mediterranean (1), Iberian Peninsula (2),Turkey (3), British Isles (4), France (5), Alps/Mid-Europe (6), Eastern Europe (7) and Scandinavia (8) a) Equal weight ($\omega_F = 0.5$, $\omega_{UF} = 0.5$) given to WSA and BSA for all cities and both land cover types (i.e. urban fabric and forest) b) BSA for all cities and land-cover types ($\omega_F = 0$, $\omega_{UF} = 0$). c) WSA for all cities and land-cover types ($\omega_F = 1$, $\omega_{UF} = 1$). d) WSA to calculate forest albedo and BSA to calculate urban fabric albedo ($\omega_F = 1$, $\omega_{UF} = 0$). e) BSA to calculate forest albedo and WSA to calculate urban fabric albedo ($\omega_F = 0$, $\omega_{UF} = 1$). f) BSA/WSA to calculate forest albedo and WSA to calculate urban fabric albedo in the regions 1,2 and 3 ($\omega_F = 0.5$, $\omega_{UF} = 1$), BSA to calculate urban fabric albedo and BSA/WSA to calculate forest albedo in the regions 4, 5, 6, 7 and 8 ($\omega_F = 0.5$, $\omega_{UF} = 0$). g) Forest albedo (equally weighed WSA/BSA) minus urban fabric WSA in regions 4,5,6 and 7 and urban fabric BSA in regions 1,2,3 and 8. h) Forest albedo (equally weighed WSA/BSA) minus urban fabric WSA in regions 4,5,6, 7 and 8 and urban fabric BSA in regions 1,2 and 3. j) Forest albedo (equally weighed WSA/BSA) minus urban fabric WSA in regions 8 and urban fabric BSA in regions 1,2, 3, 4, 5, 6 and 7.

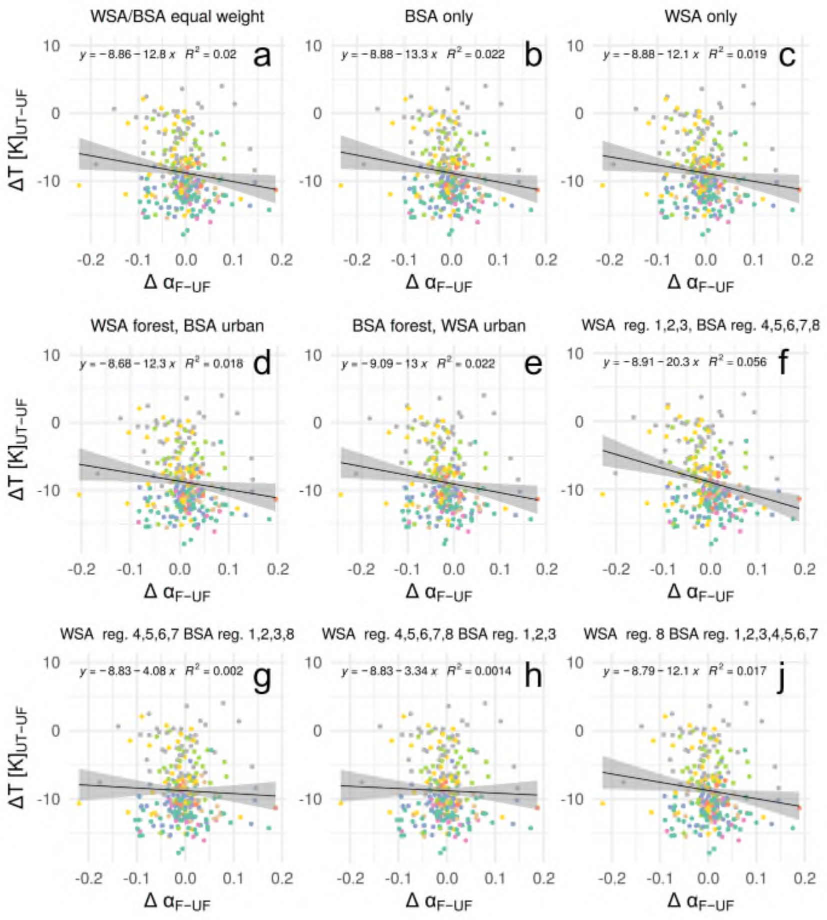

Supplementary Note 6: Selection of cities displayed in Figure 2.

For specific cities we display seasonal trends and differences between average summertime and hot extreme conditions (Figure 2). The cities were selected as being representative for all eight regions we divided Europe into. As an indicator of how well a city represents the cooling signal observed within a region, we ranked each city according to how close it was to 1. average amount of cooling provided by urban trees during summer 2. the cooling provided during hot extremes and 3. the difference between cooling during hot extremes and average summertime conditions. Based on this ranking we selected three cities in each region that closely assemble the cooling signals and among these we selected the city with the largest population. In addition, we included the city Gaziantep (Turkey) as an example for a city in which LSTs of urban tree areas are lower than LSTs of urban fabric areas.

References Supplementary Material

1. Copernicus. Mapping Guide v4.7 for a European Urban Atlas. (2016).

2. Geoportal, Basel-Stadt. Tree height based on Lidar. https://www.geo.bs.ch/, (2019).

3. Cornes RC, van der Schrier G, van den Besselaar EJM, Jones PD. An Ensemble Version of the E-OBS Temperature and Precipitation Data Sets. *Journal of Geophysical Research: Atmospheres* **123**, 9391-9409 (2018).

4. Coutts AM, White EC, Tapper NJ, Beringer J, Livesley SJ. Temperature and human thermal comfort effects of street trees across three contrasting street canyon environments. *Theoretical and Applied Climatology* **124**, 55-68 (2016).

5. Berger C, Rosentreter J, Voltersen M, Baumgart C, Schmullius C, Hese S. Spatio-temporal analysis of the relationship between 2D/3D urban site characteristics and land surface temperature. *Remote Sensing of Environment* **193**, 225-243 (2017).

6. Logan TM, Zaitchik B, Guikema S, Nisbet A. Night and day: The influence and relative importance of urban characteristics on remotely sensed land surface temperature. *Remote Sensing of Environment* **247**, 25 (2020).

7. Wu ZF, Yao L, Ren Y. Characterizing the spatial heterogeneity and controlling factors of land surface temperature clusters: A case study in Beijing. *Building and Environment* **169**, 10 (2020).

8. Chun B, Guhathakurta S. Daytime and nighttime urban heat islands statistical models for Atlanta. *Environment and Planning B: Urban Analytics and City Science* **44**, 308-327 (2017).

9. Wang D, Liang S, He T, Yu Y, Schaaf C, Wang Z. Estimating daily mean land surface albedo from MODIS data. *Journal of Geophysical Research: Atmospheres* **120**, 4825-4841 (2015).
